# Ultrasound findings and specific intrinsic blood volume expansion therapy for neonatal capillary leak syndrome: report from a multicenter prospective self-control study

**DOI:** 10.1186/s40001-024-01738-2

**Published:** 2024-03-01

**Authors:** Jing Liu, Yue-Qiao Gao, Wei Fu

**Affiliations:** 1 Department of Neonatology and NICU, Beijing Obstetrics and Gynecology Hospital, Capital Medical University, Beijing, 100026 China; 2 Beijing Maternal and Child Health Care Hospital, Beijing, 100026 China; 3 Department of Neonatology and NICU, Beijing Chao-Yang Hospital, Capital Medical University, Beijing, 100043 China; 4 Department of Neonatology and NICU, Beijing Chao-Yang District Maternal and Child Health Care Hospital, Beijing, 100026 China

**Keywords:** Capillary leak syndrome, NaCl, Hyperosmotic, Intrinsic blood volume expansion, Neonate, Clinical characteristics, Visceral edema, Point-of-care ultrasound

## Abstract

**Objective:**

Capillary leak syndrome (CLS) is characterized by severe systemic edema without specific treatment, resulting in a high mortality rate. This study investigated whether there is organ edema in neonatal CLS patients and specific treatment strategies to improve patient prognosis.

**Methods:**

Thirty-seven newborns diagnosed with CLS were included in this study. (1) Routine point-of-care ultrasound (POCUS) was used to identify whether the patients had visceral edema or fluid collection. (2) All patients were treated with 3% NaCl intravenously, and the clinical manifestations, laboratory indices and outcomes were compared before and after treatment.

**Results:**

(1) Diffuse severe edema was found in 92.0% of the patients. (2) The POCUS examination revealed that CLS patients exhibited significant visceral edema in addition to diffuse severe edema, which included pulmonary edema in 67.6%, cerebral edema in 37.8%, severe intestinal edema in 24.3%, severe myocardial edema in 8.1%, pericardial effusion in 5.4%, pleural effusion in 29.7% and peritoneal effusion in 18.9%. Two patients (5.45%) had only myocardial edema without other manifestations. (3) Before and after the intravenous injection of 3% NaCl, there were no significant differences in the serum sodium or potassium levels of CLS patients, while the hemoglobin and hematocrit levels were significantly lower after treatment (*p* < 0.01). Her plasma ALB concentration and arterial pressure returned to normal levels after the treatment was completed. (4) All the patients survived, and no side effects or complications were observed during or after treatment with 3% NaCl.

**Conclusions:**

(1) In addition to diffuse severe edema, visceral edema and effusion are common and important clinical manifestations of neonatal CLS and need to be detected by routine POCUS. (2) The intravenous injection of 3% NaCl is a safe, effective and specific treatment strategy for neonatal CLS, with a survival rate of 100% and no adverse effects.

## Introduction

Capillary leak syndrome (CLS) is a rare, potentially life-threatening systemic disease caused by increased vascular permeability due to cytokine damage to the endothelium. This disease causes the intravascular fluid and proteins to shift into the interstitial space, with subsequent hypovolemic hypotension and shock [1–3]. Generally, the major clinical characteristics of CLS include diffuse severe edema, hypovolemia or hypovolemic shock, hypoproteinemia and hemoconcentration [1–3]. Because decreased urine volume and edema are the first and most important clinical manifestations, CLS is often misdiagnosed as renal failure [2]. With the improvement of understanding of the disease, an increasing number of CLS patients have been diagnosed, and the incidence of CLS has been reported to account for 1.62% of hospitalized critically ill neonates [4]. Even so, CLS is still diagnosed on the basis of the clinical manifestations mentioned above, which can be detected by the "naked eye". Because lung ultrasound (LUS) is routinely performed in our department and has replaced chest X-ray for the diagnosis of neonatal lung diseases [5, 6], we found that some infants with CLSs have severe pulmonary edema or pleural effusion. Therefore, point-of-care ultrasound (POCUS) was routinely performed to detect whether CLS patients had edema or effusion in other organs.

Among the treatment strategies for CLS, intravenous infusion of 6% hydroxyethyl starch has long been the most common treatment; however, this approach is effective only for mild and early-stage CLS and not for severe or late-stage CLS. The mortality rate of CLSs has remained above 30% and is as high as 50% [4, 7]; therefore, it has become important for clinicians to explore therapeutic measures that can effectively reduce the mortality of CLSs without obvious side effects.

In May 2017, we diagnosed an infant with severe CLS whose disease worsened despite treatment with 6% hydroxyethyl starch. Based on the pathophysiological mechanism of the disease, we hypothesized that an intravenous hypertonic solution might have some effect. Therefore, with the informed consent of the parents, the patient was given 3% NaCl intravenously followed by furosemide. The patient showed significant improvement, with a significant increase in urine volume, a gradual decrease in edema, and eventual recovery and discharge from the hospital. Therefore, we carried out this prospective study to investigate and observe the efficacy and side effects or complications of 3% NaCl combined with furosemide for the treatment of neonatal CLS.

## Methods

### Patients

A total of 37 CLS patients diagnosed between March 2017 and December 2022 were included in this study. CLS was diagnosed according to the following criteria [1–4, 7, 8]: (1) definite cause or original etiology: severe infection, severe fetal distress or birth asphyxia, respiratory distress syndrome (RDS) or meconium aspiration syndrome (MAS); and (2) typical clinical manifestations: diffuse systemic severe edema, hypovolemia or hypovolemic shock, hypoproteinemia, hemoconcentration, a significant reduction in or the absence of urine, and significant weight gain within a short period of time. The exclusion criteria for patients were diffuse systemic edema simply due to heart disease, liver disease, kidney disease, inherited metabolic disease, or congenital malformation.

### POCUS examination

All patients underwent ultrasound examinations at any time when necessary, including brain, heart, lung, thoracic cavity, liver, kidney, and abdomen ultrasound, and the results were recorded in detail. A Voluson S10 (GE Healthcare, USA) or Philips EPIQ 5 (Philips, Netherlands) ultrasound system was used for the examination.

### Treatment strategies

#### Etiological or supportive treatment

Treatment of the primary underlying causes included the use of antibiotics if severe infection was present, mechanical ventilation if severe dyspnea was present, heparin if disseminated intravascular coagulation was present, intravenous nutritional support if there was feeding difficulty, and supplementation with alkaline preparations if severe metabolic acidosis was present.

#### Intrinsic blood volume expansion treatment

Three percent NaCl was intravenously injected according to the following methods or procedures: (1) 3% NaCl was intravenously injected at a dosage of 3–5 ml/kg within 10–20 min; (2) injections were repeated every 6 for patients with more severe edema or every 8 h for patients with relatively mild edema; and (3) as the degree of edema decreased, the interval between intravenous 3% NaCl administration gradually increased to 8-h, 12-h, and 24-h intervals and then discontinued. The administration interval was extended throughout the treatment without reducing the dose administered.

#### Intravenous injection of furosemide

Furosemide was intravenously injected into patients with severe edema (1.0 mg/kg) or relatively mild edema (0.5 mg/kg) 30 min after each injection of 3% NaCl to promote excessive fluid excretion through the kidney.

#### Pleural puncture or peritoneal puncture

Pleural puncture or peritoneal puncture was performed for 3 patients with severe pleural effusion or peritoneal effusion, among whom 1 infant underwent three successive peritoneal punctures, with more than 100 mL of peritoneal effusion extracted. This procedure removes liquid from the pleural and peritoneal cavities.

### Main outcome measures

The main outcome measures were as follows: (1) changes in serum sodium (Na^+^,mmol/L), potassium (K^+^, mmol/L), hematocrit (HCT,%), hemoglobin (Hb,g/L) and plasma albumin (g/L) levels, as well as blood pressure (mmHg) changes before and after treatment; (2) the comparison of urine volume and body weight before and after treatment; (3) the cured rate or mortality rate.

### Statistical analysis

The Statistical Package for the Social Sciences (SPSS) 24.0 software was used to statistically analyze the data. A normality test was performed before comparing the difference of the data in each group, the parametric t test was used to compare the mean values of the two groups for data that were normally distributed, while the Wilcoxon signed ranks test was used for those were not normally distributed. The cured discharge or mortality rate is expressed as a percentage. A value of *p* < 0.05 indicated a statistically significant difference.

## Results

### Demographic data and general information

Among the 37 CLS patients, 21 were boys (56.8%) and 16 were girls (43.2%). Twenty (54.1%) of the patients were delivered by cesarean section, and 17 (45.9%) were delivered vaginally. There were 25 premature infants (67.6%) and 12 full-term infants (32.4%). The average gestational age was 33.7 ± 2.6 weeks (range from 27^+5^ to 40 weeks). The average birth weight was 2280 ± 740 g (range from 900 to 3580 g).

### Etiologies and clinical manifestations

#### Etiologies

The causes of CLS included severe infection, sepsis or disseminated intravascular coagulation (DIC) in 17 patients (45.9%), RDS or MAS in 10 patients (32.4%), and severe fetal distress and birth asphyxia in 8 patients (21.6%).

#### Diffuse severe edema

The main and first clinical manifestation was diffuse severe edema, which was found in 34 of the patients (92.0%), suggesting that not all CLS patients presented with severe systemic edema.

#### Ultrasound findings

By the routine examination of point-of care ultrasound (POCUS), we found pulmonary edema in 25 patients (67.6%), cerebral edema in 14 patients (37.8%), severe intestinal edema in 9 patients (24.3%), severe myocardial edema in 3 patients (8.1%),pleural effusion in 11 patients (29.7%), peritoneal effusion in 7 patients (18.9%) and pericardial effusion in 3 patients (8.1%). Two patients (5.4%) did not have diffuse edema but presented with severe myocardial edema. One patient (2.7%) did not have diffuse edema but presented with severe cerebral edema, pulmonary edema and pleural effusion. Severe liver edema occurred in 1 patient (2.7%), and the liver was markedly swollen and enlarged, protruding into the pelvic cavity, with a hard texture and peritoneal effusion. The ultrasound diagnostic criteria for visceral edema are list in Table 1.

**Table 1:** Ultrasound diagnostic criteria for visceral edema

Organ edema	Diagnostic criteria
Pulmonary [9, 10]	Significantly increased B-lines, confluent B-lines, compact B-lines or white lung
Cerebral [11–13]	(1) Diffuse or focal brain parenchymal increased echogenicity. (2) Lateral ventricle narrowed, disappeared or slit-shaped. (3) Effacement of the cerebral sulci. (4) The pulsation of cerebral arteries decreased or disappeared
Intestinal [14]	(1) Intestinal wall was significantly thickened. (2) Except for other causes
Myocardial [15]	(1) The thickness of myocardium significant increased. (2) The echo of myocardium decreased. (3) The diameter of heart chamber significantly decreased. (4) Except for other causes

Hypoproteinaemia, hypovolemia or hypovolemic shock, and hemoconcentration were observed in all of the patients.

There were significant reductions in urine volume and weight gain in 34 (92.0%) of the patients.

### Treatment effects and outcomes

#### Effects of 3% NaCl treatment on the arterial pressure of CLS patients

The systolic blood pressure (SBP, mmHg), diastolic blood pressure (DBP, mmHg) and mean arterial blood pressure (MBP, mmHg) were restored to normal values in all CLS patients after treatment with 3% NaCl (Table 2).

**Table 2:** Influence of 3% NaCl treatment on blood pressure (mmHg, *x̄* ± s)

	SBP	DBP	MBP
Before treatment	53.1 ± 5.47	25.5 ± 4.45	39.3 ± 4.44
After treatment	64.1 ± 5.84	37.0 ± 5.56	50.6 ± 5.20
*t/z-*value	5.145	9.511	9.743
*p-*value	0.000	0.000	0.000

1 mmHg = 0.133 kPa

SBP: non-normal distribution, Wilcoxon signed ranks test

#### Effects of 3% NaCl treatment on serum sodium, serum potassium, hemoglobin, hematocrit, and plasma albumin levels, urine volume and body weight in CLS patients

After 3% NaCl treatment, the body weight decreased significantly along with a significant increase in urine volume, the plasma albumin level increased significantly and returned to normal, the hemoglobin concentration and hematocrit level decreased significantly, and there was no significant change in serum sodium and potassium levels before and after treatment, no infants developed hypernatremia after 3% NaCl treatment (Table 3).

**Table 3:** Effects of 3% NaCl treatment on serum sodium, serum potassium, hemoglobin, hematocrit, and plasma albumin levels, urine volume and body weight in CLS patients (*x̄* ± *s*)

	Na^+^ (mmol/L)	K + (mmol/L)	ALB (g/L)	HCT (%)	Hb (g/L)	UV [mL/(kg·h)]	Wt (g)
Before	137.7 ± 3.00	4.25 ± 0.49	22.7 ± 3.68	38.5 ± 5.17	137.0 ± 19.4	2.61 ± 0.9	2591 ± 687
After	138.2 ± 2.64	4.22 ± 0.53	30.2 ± 5.80	33.6 ± 4.72	118.5 ± 15.3	5.68 ± 1.0	2370 ± 657
*t/z-value*	0.803	1.310	4.603	4.775	4.719	13.847	7.365
*p value*	0.425	0.182	0.000	0.000	0.000	0.000	0.000

ALB: albumin; HCT: hematocrit; Hb: hemoglobin; UV: urine volume; Wt: weight

ALB, HCT and Hb: non-normal distribution, Wilcoxon signed ranks test

#### Outcomes

All 37 CLS patients were clinically cured at discharge; thus, the survival rate was 100%, that is, the fatality rate was 0%.

## Discussion

According to the results of the present study, the major clinical characteristics of neonatal CLSs are as follows: (1) the most common causes are severe infection and sepsis, as well as RDS, and the least common causes are severe asphyxia and MAS. (2) The most common clinical manifestations are diffuse severe edema, decreased urine volume, rapid weight gain, hypovolemia or hypovolemic shock, hypoalbuminemia and hemoconcentration [1, 2, 16]. Therefore, CLS is often misdiagnosed as hypovolemic shock, septic shock, polycythemia vera, or angioedema [17, 18]. (3) POCUS reveals internal organ edema or effusion, including cerebral edema, pulmonary edema, myocardial edema, intestinal edema and pleural or abdominal fluid accumulation or pericardial effusion, as common manifestations of CLS, even in the small number of infants with only organ edema as their main manifestation. Although brain damage and lung damage have been described in CLS patients [19, 20], this study is the first to identify severe heart damage, liver damage, and intestinal damage in CLS patients by ultrasound examination. Without ultrasound, these manifestations are difficult to detect and may result in improper management and a poor prognosis. Therefore, routine ultrasound examination is highly valuable for patients with suspected or high-risk factors.

The pathogenesis of CLS has not been completely elucidated [21–24]. It is generally believed that capillary endothelial damage and increased permeability of endothelial cells are caused by various factors, resulting in leakage of water and albumin from the plasma into the tissue space [21–24]. During the early phase, only small molecular weight substances extravasate into the tissues, and the fluid and electrolytes then enter the tissue space. As the disease progresses, relatively large molecular weight substances such as albumin can also leak out; thus, the tissue colloidal osmotic pressure increases, and the fluid extravasates into the tissue space. Consequently, severe diffuse edema and a decrease in the effective circulating blood volume occur, resulting in hypotension and injury to systemic tissues and organs [25].

Therefore, effectively increasing blood volume and maintaining a normal systemic blood supply and blood pressure are critical for successful treatment of this condition. During the early stages, 0.9% NaCl is generally not the first choice due to the increased capillary permeability [1, 8, 16]. Artificial colloids, such as hydroxyethyl starch, have a high molecular weight and an electrolyte composition similar to that of plasma. They can not only maintain colloidal osmotic pressure but also supplement extracellular electrolyte and alkali reserves, maintain effective renal perfusion, reduce the risk of oliguria or anuria, reduce the release of inflammatory mediators, decrease inflammatory reactions and reduce endothelial injury. In addition, they can plug capillaries with increased permeability, reduce plasma viscosity and improve tissue oxygen supply. Therefore, they are commonly used for volume expansion during CLS leakage stages [1, 8, 16, 26]. However, the literature and related experience have shown that this treatment is effective only for infants with mild conditions in the early phases, and more severe patients still have a mortality rate of more than 30% [4, 7]. Therefore, new treatment methods should be introduced to improve the cure rate and prognosis of infants with CLS and shorten the treatment period.

In the present study, we used 3% NaCl to treat neonatal CLS patients and obtained good results and excellent outcomes. All of the patients were cured and discharged, with a survival rate of 100% and a mortality rate of 0%, and there were no electrolyte disorders, such as hypernatremia or hypokalemia. With increasing urine volume, the body weight decreased gradually, the plasma ALB concentration and arterial pressure returned to normal levels, and the Hb and hematocrit levels improved.

The mechanism of the combination of 3% NaCl and furosemide in the treatment of CLS has not been well documented. However, according to our knowledge and clinical experience, the following factors may be involved. During the leakage stage of CLS, the infusion of 3% NaCl increased plasma osmolality by more than threefold in a short period. The effective ion concentration gradient produced by 3% NaCl reabsorbs the liquid that leaks into the tissue space from the blood circulation by the hyperosmotic effect, expanding the blood volume and stabilizing the arterial pressure without increasing the total amount of liquid within the body. This approach*** to increasing blood volume is referred to as "*intrinsic blood volume expansion*" in this study. With the application of 3% NaCl, the interstitial fluid can return to the blood vessels to maintain an effective circulating blood volume. As the blood volume is restored, the hemoconcentration caused by volume reduction is restored to its proper state. Intravenous furosemide was given to patients after the above effects were achieved; this agent quickly drained the excess fluid in the blood vessels out of the body, not only reducing edema but also preventing aggravation of the heart load due to a significant increase in blood volume. Moreover, with the introduction of hypertonic sodium chloride into the circulation, the crystalloid osmolality of plasma increases while the colloid osmolality is relatively low, and the colloid osmolality gradient between blood and tissue fluid also changes. To maintain the osmotic equilibrium of the new colloid, the albumin that had leaked into the interstitial space returned to the plasma, after which the plasma ALB concentration was subsequently normalized.

Diffuse severe edema is often the first clinical manifestation to be observed by clinicians, but according to our clinical experience, decreased blood pressure or hypotension often precedes edema. When CLS is still in the early stage and other symptoms are not obvious, clinicians often use 0.9% NaCl infusion or even combination NaCl with vasoactive agents such as dopamine. However, this inappropriate treatment not only has difficulty correcting hypotension but also may aggravate circulatory disorders and even worsen the patient’s condition. Likewise, albumin supplements should not be given to CLS patients. Although intravenous injection of 25% albumin is still widely recommended [1, 3], both our clinical experience and the literature suggest that it quickly leaks into the interstitial space and further exacerbates edema in patients [27]. After recovery, the infant's plasma ALB concentration can naturally return to normal levels.

Some limitations of this treatment approach were also identified. First, the optimal dosage and duration of treatment are not well established and may vary depending on the severity of the disease and individual patient factors. Second, the long-term outcomes of this treatment are unknown and require further investigation. Finally, the study was limited by its small sample size and lack of a control group. However, due to the high efficacy and safety of this treatment regimen, these limitations do not affect its use in clinical practice.

## Conclusions

In conclusion, the results of this study indicate the following: (1) Visceral edema and effusion are common and important clinical manifestations of neonatal CLS. Therefore, CLS patients or those with high risk factors for CLS should be routinely monitored via point-of-care ultrasound. (2) The adjuvant treatment of neonatal CLSs with 3% NaCl is a very effective treatment strategy without side effects or complications, even though the optimal dosage and duration of treatment are not well established; further research is needed to confirm the long-term outcomes of this treatment approach.

## Data Availability

The dataset used and analyzed is available from the corresponding author upon reasonable request.

## References

[1] Bichon A, Bourenne J, Gainnier M, Carvelli J (2021). Capillary leak syndrome: state of the art in 2021. Rev Med Interne.

[2] Bozzini MA, Milani GP, Bianchetti MG, Fossali EF, Lava SAG (2018). Idiopathic systemic capillary leak syndrome (Clarkson syndrome) in childhood: systematic literature review. Eur J Pediatr.

[3] Siddall E, Khatri M, Radhakrishnan J (2017). Capillary leak syndrome: etiologies, pathophysiology, and management. Kidney Int.

[4] Sheng LJ, Zhao HY, Ding Y, Huang WM (2017). Epidemiology investigation on capillary leakage syndrome in critically ill newborns. Chin Pediatric Emerg Med.

[5] Gao YQ, Qiu RX, Liu J, Zhang L, Ren XL, Juan Qin SJ (2022). Lung ultrasound completely replaced chest X-ray for diagnosing neonatal lung diseases: a 3-year clinical practice report from a neonatal intensive care unit in China. J Matern Fetal Neonatal Med.

[6] Liu J, Zhang X, Wang Y, Li J, Yan W, Qin SJ (2022). The outcome- or cost-effectiveness analysis of LUS-based care or CXR-based care of neonatal lung diseases: the clinical practice evidence from a level III NICU in China. Diagnostics.

[7] Wei H, Zhang ZY, Li LQ (2020). Risk factors in the prognosis of neonatal capillary leak syndrome. J Clin Pediatr.

[8] Baloch NUA, Bikak M, Rehman A, Rahman O (2018). Recognition and management of idiopathic systemic capillary leak syndrome: an evidence-based review. Expert Rev Cardiovasc Ther.

[9] Liu J, Copetti R, Sorantin E, Lovrenski J, Rodriguez-Fanjul J, Kurepa D (2019). Protocol and guidelines for point-of-care lung ultrasound in diagnosing neonatal pulmonary diseases based on international expert consensus. J Vis Exp.

[10] Liu J, Chen XX, Li XW, Wang Y, Chen SW, Fu W (2016). Lung ultrasonography to diagnose transient tachypnea of the newborn. Chest.

[11] Barr LL (1999). Neonatal cranial ultrasound. Radiol Clin North Am.

[12] Maller VV, Cohen HL (2019). Neonatal head ultrasound: a review and update-Part 1: techniques and evaluation of the premature neonate. Ultrasound Q.

[13] Maller VV, Choudhri AF, Cohen HL (2019). Neonatal head ultrasound: a review and update-Part 2: the term neonateand analysis of brain anomalies. Ultrasound Q.

[14] van Wassenaer EA, de Voogd FAE, van Rijn RR, van der Lee JH, Tabbers MM, van Etten-Jamaludin FS (2020). Bowel ultrasound measurements in healthy children—systematic review and meta-analysis. Pediatr Radiol.

[15] Liu J, Gao YQ (2023). Myocardial edema: a rare but specific manifestation of neonatal capillary leak syndrome. Diagnostics.

[16] Nong SH (2020). Neonatal capillary leak syndrome. Zhongguo Dang Dai Er Ke Za Zhi.

[17] Eo TS, Chun KJ, Hong SJ, Kim JY, Lee IR, Lee KH (2018). Clinical presentation, management, and prognostic factors of idiopathic systemic capillary leak syndrome: a systematic review. J Allergy Clin Immunol Pract.

[18] Raith EP, Ihle JF, Jamieson J, Kalff A, Bosco J (2018). Idiopathic systemic capillary leak syndrome presenting as septic shock: a case report. Heart Lung.

[19] Yamagami K, Miyaichi T, Kanki R (2021). Cerebral involvement in systemic capillary leak syndrome. Intern Med.

[20] Bahloul M, Ketata W, Lahyeni D, Mayoufi H, Kotti A, Smaoui F (2021). Pulmonary capillary leak syndrome following COVID-21 virus infection. J Med Virol.

[21] Xie Z, Chen WS, Yin Y, Chan EC, Terai K, Long LM (2018). Adrenomedullin surges are linked to acute episodes of the systemic capillary leak syndrome (Clarkson disease). J Leukoc Biol.

[22] Xie Z, Ghosh CC, Patel R, Iwaki S, Gaskins D, Nelson Jones N (2012). Vascular endothelial hyperpermeability induces the clinical symptoms of Clarkson disease (the systemic capillary leak syndrome). Blood.

[23] Sek AC, Xie Z, Terai K, Long LM, Nelson CN, Dudek AZ (2015). Endothelial expression of endothelin receptor A in the systemic capillary leak syndrome. PLoS ONE.

[24] Xie Z, Kuhns DB, Gu X, Otu HH, Libermann TA, Gallin JI (2019). Neutrophil activation in systemic capillary leak syndrome (Clarkson disease). J Cell Mol Med.

[25] Siddall E, Radhakrishnan J (2019). Capillary leak syndrome: a cytokine and catecholamine storm?. Kidney Int.

[26] Qu Y, Tang W, Hao M, Chen X (2021). A preliminary study of influences of hydroxyethyl starch combined with ulinastatin on degree of edema in newborns with capillary leak syndrome. Am J Transl Res.

[27] de Chambrun MP, Luyt CE, Beloncle F, Gousseff M, Mauhin W, Argaud L (2017). The clinical picture of severe systemic capillary-leak syndrome episodes requiring ICU admission. Care Med.

